# PDBe: improved findability of macromolecular structure data in the PDB

**DOI:** 10.1093/nar/gkz990

**Published:** 2019-11-06

**Authors:** David R Armstrong, John M Berrisford, Matthew J Conroy, Aleksandras Gutmanas, Stephen Anyango, Preeti Choudhary, Alice R Clark, Jose M Dana, Mandar Deshpande, Roisin Dunlop, Paul Gane, Romana Gáborová, Deepti Gupta, Pauline Haslam, Jaroslav Koča, Lora Mak, Saqib Mir, Abhik Mukhopadhyay, Nurul Nadzirin, Sreenath Nair, Typhaine Paysan-Lafosse, Lukas Pravda, David Sehnal, Osman Salih, Oliver Smart, James Tolchard, Mihaly Varadi, Radka Svobodova-Vařeková, Hossam Zaki, Gerard J Kleywegt, Sameer Velankar

**Affiliations:** 1 Protein Data Bank in Europe, European Molecular Biology Laboratory, European Bioinformatics Institute (EMBL-EBI), Wellcome Genome Campus, Hinxton, Cambridge CB10 1SD, UK; 2 CEITEC - Central European Institute of Technology, Masaryk University Brno, Kamenice 5, 625 00 Brno-Bohunice, Czech Republic; 3 InterPro, European Molecular Biology Laboratory, European Bioinformatics Institute (EMBL-EBI), Wellcome Genome Campus, Hinxton, Cambridge CB10 1SD, UK; 4 Electron Microscopy Data Bank (EMDB), European Molecular Biology Laboratory, European Bioinformatics Institute (EMBL-EBI), Wellcome Genome Campus, Hinxton, Cambridge CB10 1SD, UK

## Abstract

The Protein Data Bank in Europe (PDBe), a founding member of the Worldwide Protein Data Bank (wwPDB), actively participates in the deposition, curation, validation, archiving and dissemination of macromolecular structure data. PDBe supports diverse research communities in their use of macromolecular structures by enriching the PDB data and by providing advanced tools and services for effective data access, visualization and analysis. This paper details the enrichment of data at PDBe, including mapping of RNA structures to Rfam, and identification of molecules that act as cofactors. PDBe has developed an advanced search facility with ∼100 data categories and sequence searches. New features have been included in the LiteMol viewer at PDBe, with updated visualization of carbohydrates and nucleic acids. Small molecules are now mapped more extensively to external databases and their visual representation has been enhanced. These advances help users to more easily find and interpret macromolecular structure data in order to solve scientific problems.

## INTRODUCTION

Protein Data Bank in Europe (PDBe; pdbe.org) is a founding member of the Worldwide Protein Data Bank (wwPDB; wwpdb.org) (1), the international consortium responsible for the management of the Protein Data Bank (PDB) (2), the single, global archive for experimentally determined three dimensional (3D) structures of biological macromolecules. The other wwPDB partners are Research Collaboratory for Structural Bioinformatics Protein Data Bank (RCSB PDB; rcsb.org) (3), Protein Data Bank Japan (PDBj; pdbj.org) (4) and Biological Magnetic Resonance Bank (BMRB; bmrb.wisc.edu) (5). Together, the four partners fully cooperate in the areas of deposition, curation, validation and dissemination of macromolecular structure data, guided by the FAIR principles of administering data resources, which ensure data is Findable, Accessible, Interoperable and Reusable (2,6).

Since 2014, the processing of all PDB depositions is managed through the OneDep system (7). PDBe is responsible for the processing of OneDep depositions from European and African institutions, totalling over 4,000 PDB entries in 2018, which equates to 34% of the total PDB depositions that year (wwpdb.org/stats/deposition). PDBe and the wwPDB partners collaborate closely with the Electron Microscopy Data Bank (EMDB) (8), the archive for electron microscopy (EM) electric potential maps, which are also deposited via OneDep. While all wwPDB partners collaborate on processing deposited data in the PDB archive, thereby creating an authoritative source of macromolecular structure data, each partner has its own website and tools to support users in accessing the data.

In 2019, the PDB reached the milestone of 150 000 structures. This wealth of information is used by researchers in fields ranging from drug discovery to protein engineering. To support these diverse user communities, and to present macromolecular structure information in other biologically relevant contexts, PDBe enriches its data through collaborations with resources that specialize in other areas of bioinformatics. For example, the SIFTS project (9) brings together PDB structures with protein sequence data and annotations from UniProtKB (10), Pfam (11) and InterPro (12), structure domains from CATH (13) and SCOP (14), and, more recently, started incorporating genetic variation data from Ensembl (15) and putative homology groups from Homologene (16). PDBe provides online data access, analysis and visualization tools for PDB data through its website, FTP/RSYNC file download, Application Programming Interface (REST API) and PDB Coordinate/Electron-density servers. PDBe services were accessed from 2.2 million unique IP addresses in 2018, with 1.1 million users accessing the PDBe REST API in the same time period, based on statistics collected at the European Bioinformatics Institute (EMBL-EBI) (17).

A new resource, Protein Data Bank in Europe-Knowledge Base (PDBe-KB, pdbe-kb.org), has been developed by PDBe in collaboration with the structural bioinformatics community. The PDBe-KB resource aggregates macromolecular structure data in a series of dedicated views centred on commonly used biological objects (e.g. UniProtKB accessions) rather than on PDB entries. These views incorporate additional biological context and functional information and are discussed in a separate publication in this issue (18).

This paper describes data enrichment efforts and updates to the PDBe infrastructure, website and other services since our previous publication (19).

## AUTOMATING THE PDBE RELEASE PROCESS

The wwPDB partners release PDB archive data through their respective websites and FTP sites at 00:00 UTC each Wednesday. PDBe have implemented a robust release process using Apache Airflow (airflow.apache.org), a free and open source software for workflow and dependency management. Airflow is used to manage job dependencies in the weekly release process, supporting automation and improving efficiency. The Airflow software enables thorough monitoring of the large number of processes that are involved in the release of data at PDBe, providing clear notification of any errors. The Airflow system includes a sophisticated graphical interface for review of the overall release process, highlighting any failed processes and allowing easy restart of individual tasks. This new infrastructure has significantly streamlined the complex process of releasing data at PDBe, ensuring timely availability of data and allowing easy integration of additional software into the PDBe release process, some of which is described below.

## IMPROVEMENTS TO SMALL-MOLECULE DEPICTION

**Figure 1.**
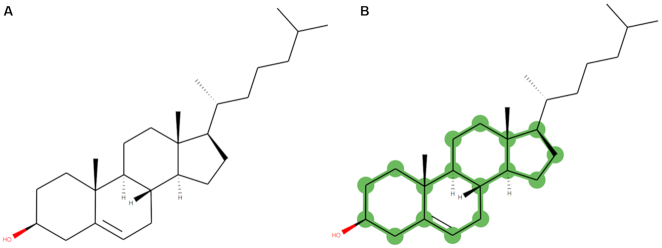
(**A**) Image of cholesterol (PDB three-letter code: CLR) generated from the PDB Chemical Component Dictionary, using the pdbeccdutils process. (**B**) Chemical scaffold (in green) of cholesterol, identified and highlighted using the pdbeccdutils process.

Previous efforts to improve the PDBe website focused on macromolecules, and the area of small molecules was identified as a future improvement (19). To support clear and effective web-delivery of information pertaining to small molecules in the PDB, we have now redesigned the underlying process for handling small molecules present in the wwPDB chemical component dictionary (CCD) (20), deriving added-value information, improving two-dimensional (2D) depictions and adding cross-references to cheminformatics resources. The redesigned process is implemented as a freely available python package (pdbeccdutils; https://gitlab.ebi.ac.uk/pdbe/ccdutils) that builds on the RDKit software (http://rdkit.org) and its data model. The package provides functionality for reading PDBx/mmCIF-formatted files (compound definitions from the CCD) as well as utility functions to compute physicochemical properties, scaffolds, fragments and to draw ‘collision-free’ images using a series of templates (Figure 1). This process generates 2D coordinates and schematic depictions for the vast majority of small molecules in the PDB (e.g. only 447 out of 29,318 compounds do not have a collision-free image as of September 2019). The images have been added to the PDBe search interface and PDB entry pages (see below). Where possible, we have added cross-references to the database entries for the same compound in other resources, including ChEMBL (21), ChEBI (22), ZINC (23) and DrugBank (24).

## DATA ENRICHMENT

Macromolecular structures provide insight into the function of biological systems, but to capitalize fully on the value of the data inherently present in the PDB, it must be presented in the context of related biological and chemical information, particularly for scientists whose expertise may be outside of structural biology. PDBe works closely with other teams at EMBL-EBI and beyond, to enrich the data in the PDB archive with additional biological and chemical information. PDBe have been involved in a number of projects that have added value to PDB entries where this information was previously absent or incomplete (Table 1). Unlike the majority of proteins, for which UniProtKB is a universally accepted reference resource for sequence information, RNA molecules in PDB entries are not mapped to any sequence database that could provide consistent molecule naming using conventions adopted by the community. For example, it is difficult to find all transfer RNA structures in the PDB. To address this issue, PDBe, in collaboration with the Rfam team at EMBL-EBI, have mapped RNA sequences to RNA families provided by the Rfam resource (25). Functional annotation of small molecules in the PDB is lacking, and to start to address this issue, PDBe has collaborated with the Thornton group to identify cofactor molecules in PDB entries (26). To address the discrepancy between the release cycles of the PDB and the Pfam and CATH resources, PDBe have integrated preliminary assignment of protein families (Pfam) and structural domains (CATHb (13)) such that these domains can be identified prior to formal assignment by Pfam and CATH (9). These data-enrichment projects are summarized in Table 1.

**Table 1.** Summary of data enrichment collaborations

Enriched data	Enrichment process	Main outcomes
Mapping to Rfam	• Data incorporated into the PDBe database and displayed on relevant PDBe entry pages. • Data integrated into the PDBe REST API, search and in visualization, via web components. • Links to Rfam resource on PDBe entry pages. • Rfam data resource was updated where missing mapping information was identified.	• Assignment of over 5000 RNA chains to 98 Rfam families in >1500 structures in the PDB. • Improved findability of RNA structures. • Easier identification of RNA function in PDB entries.
Identification of cofactors	• PDBe worked with the Thornton group at EMBL-EBI to set up a process to identify cofactor and cofactor-like molecules in the PDB. • Cofactors grouped by class. • The process verifies that such molecules are bound to enzymes associated with the correct cofactor class. • Data made available via PDBe REST API, on PDBe entry pages and search.	• Identified 417 unique cofactor-like small molecules. • Annotation of over 78 000 PDB entries containing enzymes, 12 000 contain cofactor or cofactor-like molecules, representing over 1500 different Enzyme Commission numbers.
Preliminary Pfam domain assignments	• Collaboration with Pfam team to implement provisional domain-assignment process at PDBe. • Pfam domains assigned in newly released PDB entries, in advance of Pfam database release. • PDBe integrates this data, enabling Pfam domain assignments for PDB entries immediately after release.	• As of September 2019, over 16 000 PDB entries had Pfam domains assigned which are not in the official latest Pfam release (V32.0, September 2018). • This includes over 3100 distinct Pfam domains.
Preliminary CATH domain assignments (CATHb)	• CATHb data released by CATH team provides preliminary CATH structural domain assignment for PDB structures on a weekly basis. • PDBe integrates this data, enabling structure-domain assignments for PDB entries immediately after release.	• As of August 2019, around 30 000 new entries have CATH domains assigned which are not in the official full CATH release (V4.2, September 2017).
Standardized information on crystallographic cells dimensions (NIGGLI)	• Standardization of cell dimensions using Niggli reduction (27). • Standardized cell dimensions made available through PDBe search API.	• Standardized cell dimensions in PDBe's search used by Phaser (28).

All the data from the above enrichment activities are made available via the PDBe REST API and are integrated into PDBe's search system.

## DATA QUALITY AND INTEGRITY

To ensure findability of data, users must be able to search with familiar terms and retrieve accurate and up-to-date results. One of the biggest barriers to findability is poor data quality and integrity. Therefore, to improve findability, PDBe strives to make the data as consistent as possible. As described previously (19), PDBe's ‘clean-mmCIF’ process updates PDBx/mmCIF files from the PDB archive with remapped enumerations and additional information, yielding more consistent, standardized metadata, without altering core PDB information, such as atomic coordinates and experimental data.

Since originally reported (19), a further 13 categories are now updated through the clean-mmCIF process, including information on synchrotron site and beamline for X-ray crystallography entries, and software information for all experimental techniques. A key aim of this process is to identify where data is inconsistent so that the core PDB archive can be improved. Many issues identified by this process have since been converted into checks or enumerations in the wwPDB OneDep system (7). Of the 25 categories updated in the clean-mmCIF process, nine have now been updated archive-wide, including experimental method and software classification, with a further five categories standardized for newly deposited entries, e.g. names of software packages used in EM and NMR.

## SEQUENCE CLUSTERING

In many cases, the PDB contains more than one structure of the same protein or domain, with the current over 155 000 PDB entries representing only 48 000 unique UniProtKB entries (https://www.ebi.ac.uk/pdbe/docs/sifts/statistics.html). It is also common to find structures of homologous proteins. In many applications, this redundancy can be reduced, especially where users of the PDB archive wish to use a single representative structure for their protein of interest. Identification of redundant protein structures can be achieved by sequence comparison, as high sequence similarity correlates with structural similarity (29). PDBe have implemented a sequence-clustering process, using the MMseqs software suite (30), that groups structures based on sequence identity at various thresholds: 100%, 95%, 90%, 70%, 50%, 40% and 30%. PDB users looking for only individual representative structures at a certain level of sequence identity can now do so by applying a sequence-clustering filter in either the PDBe REST API or the PDBe search interface. This results in only individual representative structures being returned for each unique sequence cluster at the specified identity threshold, thus reducing the bulk of search results.

## IMPROVED QUERY SYSTEM

To ensure that data can be accessed effectively by a variety of user groups, it is vital that data of interest can be easily discovered in a format that is simple to interpret. In 2015, PDBe introduced a new query system (31), providing innovative and intuitive search options for accessing PDB data. More recent developments have focused on further improving the findability of macromolecular structures at PDBe, both by improving the data and by building on the existing search functionality.

### Improved autocomplete

The autocomplete functionality introduced previously at PDBe (19) has streamlined the querying of PDB data, while still presenting a large number of search terms. As data has been enriched with new mappings (Table 1), they have been included as new search items. The addition of more search items often expanded the autocomplete suggestions beyond a single page view, thereby reducing its usability. To mitigate this issue, the categories have been grouped into fewer sections. For example, rather than having separate groups for sequence and structural domains, these have been grouped into a common domains group, including Pfam, InterPro, CATH, SCOP and Rfam, simplifying the display of the autocomplete suggestions.

### Advanced search

**Figure 2.**
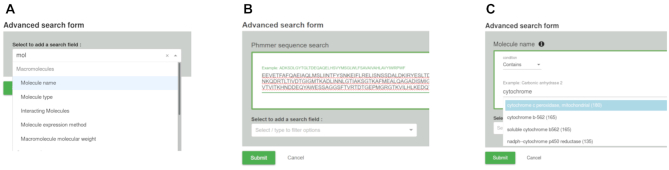
New features added to the PDBe search. (**A**) The advanced search form supports 122 fields in queries. (**B**) Sequence searching using HMMER. (**C**) Autocomplete option to find relevant search terms for each search field.

PDBe has increased the number of data categories available for searching to 122, enabling users to create richer and more customized queries. All search categories are now available in a new ‘Advanced search’ form (pdbe.org/search). They are grouped into sections containing related terms, with an autocomplete option also available to facilitate identification of relevant search fields (Figure 2). Furthermore, autocomplete has been implemented within each search category to simplify identification of relevant search terms and improve findability. Thirty new experimental data items have been added to the search related to EM and NMR structure determination methodologies. The ‘resolution revolution’ in EM (32) has significantly raised the interest in this method and these additional categories help users to identify structures determined with particular experimental procedures. The accuracy of the data in these additional data categories has improved by the development of OneDep and enumerations in the PDBx/mmCIF dictionary, in addition to clean-up efforts by wwPDB biocurators, ensuring consistent and accurate capture of data in PDB depositions (2,7,33).

In search fields containing numeric and date terms, the form allows users to set specific ranges to fine-tune search queries. Each text-based term can be searched with options including ‘contains’ and ‘equal to’, and multiple search terms can be linked with Boolean operators (‘AND’, ‘OR’, and ‘NOT’).

**Figure 3.**
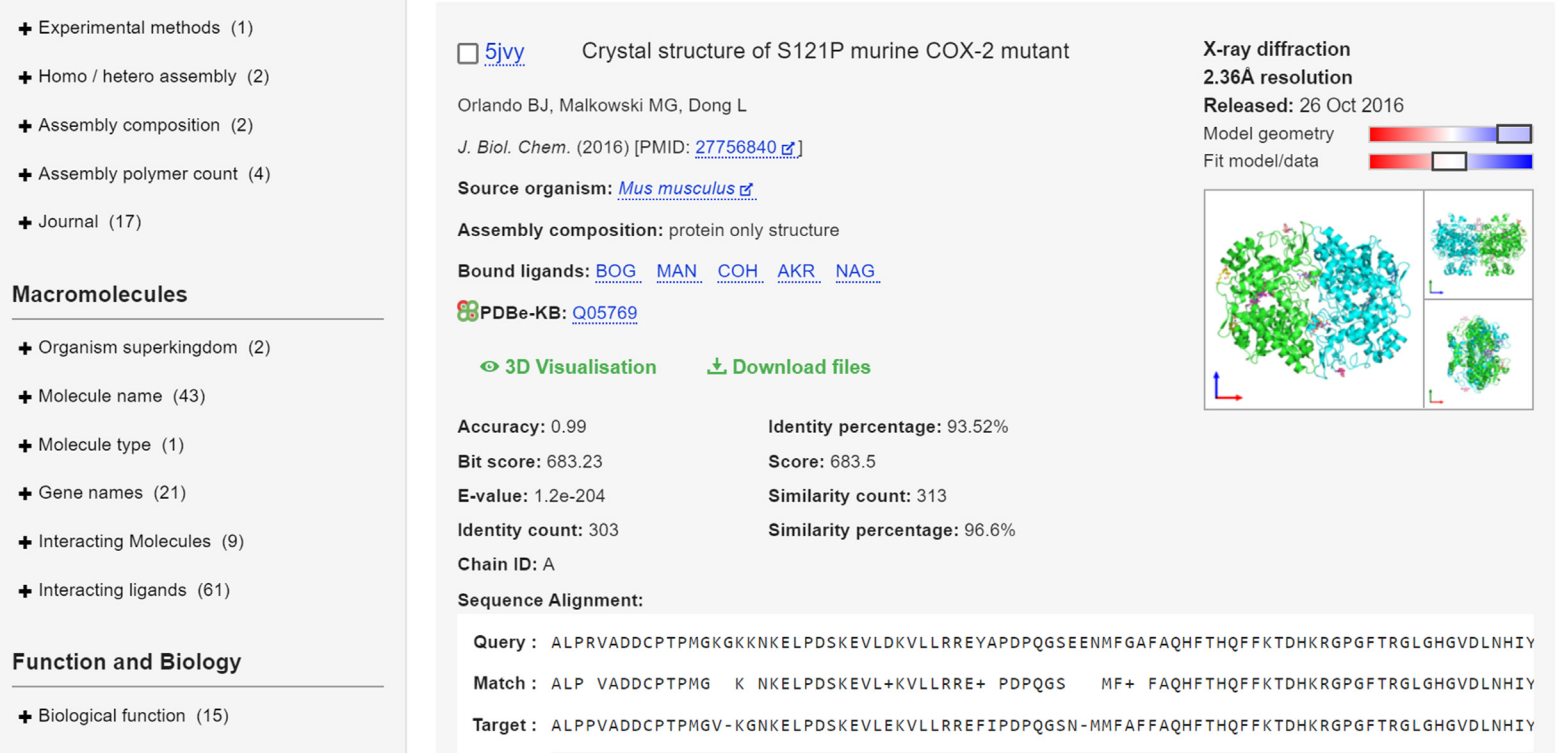
New features in presenting search results, including: number of facets (left) increased to 39 to improve filtering of results, access to 3D visualization and file downloads directly from the search results (green text); alignment and statistics provided for sequence searches, links to PDBe-KB aggregated views.

Whether the query is submitted via the simple or advanced search interface, the user is offered further options to download the search results or filter them with facets (Figure 3). Direct links for 3D visualization and additional downloads for each search result allow faster access to the PDB structures and further information. There are links to the relevant protein-centric aggregated views served by PDBe-KB (pdbe-kb.org/proteins) for each protein, giving direct access to the wealth of additional information provided by that resource (18).

The ‘Macromolecules’ tab in the PDBe search results page lets users see their results grouped by individual molecule name, presenting a more succinct view of the available data. This feature was limited to grouping by protein name, but now includes grouping by DNA and RNA molecule names.

### Sequence search

There are numerous services available to search for proteins based on sequence similarity. Tools such as BLAST (34), FASTA (35), and HMMER (36) enable users to easily query very large databases of protein sequences and obtain meaningful results rapidly. Though these tools may provide links to PDB entries, they are usually presented as a list of accession codes and do not provide additional context and validation information for the search results. PDBe have integrated sequence searching with FASTA into the advanced search form, in addition to querying with the EMBL-EBI pHMMER service (www.ebi.ac.uk/Tools/hmmer/search/phmmer) which carries out fast and sensitive homology searches using protein sequence profiles. Results are initially sorted by similarity score and can be further refined with PDBe search facets or sorted by other criteria such as validation score or resolution.

## ADDITIONAL DATA FOR BULK ACCESS

As a wwPDB member, PDBe hosts copies of both the PDB FTP site (ftp://ftp.ebi.ac.uk/pub/databases/pdb) and the PDB versioned FTP site (http://ftp.ebi.ac.uk/pub/databases/pdb_versioned/), providing FTP/RSYNC access to PDB data. Enriched data unique to PDBe is provided by the PDBe REST API (pdbe.org/api), which powers both the PDBe search and its entry pages, and is distributed through an additional FTP site (ftp://ftp.ebi.ac.uk/pub/databases/msd). The data available from both the REST API and PDBe FTP site are updated to reflect changes and improvements to the PDBe website, with recent updates detailed in Table 2.

**Table 2.** Additions and updates to the PDBe REST API and FTP sites

PDBe REST API (pdbe.org/api)	• New endpoints for protein information. ∘ CATHb structure domains. ∘ Pre-release Pfam sequence domains (via HMMER). ∘ Ensembl identifiers. • New endpoints for nucleic-acid information. ∘ RNA molecules mapped to Rfam. • New endpoints for information on small molecules in the Chemical Component Dictionary (CCD). ∘ Cofactor information. ∘ Cross-references to DrugBank (including UniProtKB accessions for drug targets), ChEBI (22), ChEMBL (21) and CSD (37).
PDBe enriched FTP (ftp://ftp.ebi.ac.uk/pub/databases/msd)	• mmCIF-format assembly files added (ftp://ftp.ebi.ac.uk/pub/databases/msd/assemblies). ∘ These contain full assemblies for a given structure, along with rotation/translation operators required to generate the assemblies from the PDB entry file. ∘ Includes XML files summarizing the composition of the assembly files, relating each chain in the assembly to an entity in the PDB entry. • New FTP area for updated chemical-component process (ftp://ftp.ebi.ac.uk/pub/databases/msd/pdbechem_v2). ∘ CCD mmCIF files with additional cross-references, mapped through the UniChem pipeline (38), to ChEMBL, DrugBank, KEGG, ChEBI, ZINC, Pubchem, NMRShiftDB, BindingDb, MetaboLights, BRENDA and Rhea. ∘ Updated image files for molecules in CCD. ∘ Coordinate data in various formats – including PDB and SDF.

## DEVELOPMENTS TO VISUALIZATION IN LITEMOL

**Figure 4.**
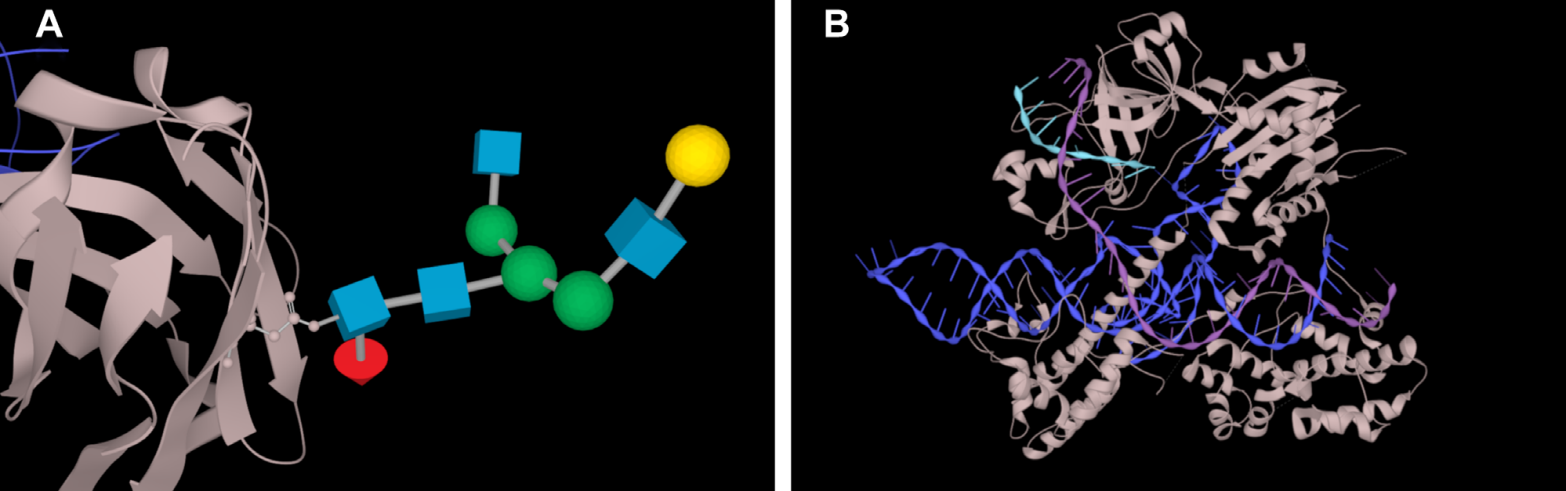
Improved visualization of nucleic acids and carbohydrates in LiteMol. (**A**) PDB entry 5ezi (48), the structure of a malaria antigen in complex with an antibody, focused on the branched glycan in the structure. The glycan is shown using the 3D-SNFG representation. (**B**) PDB entry 5x2g (49), structure of a CRISPR-Cas9 complex of protein with nucleic acids, using the new visualization for RNA and DNA.

The LiteMol viewer (39), has enabled fast visualization of PDB structures and related functional data at PDBe. Several features that further improve the representation of PDB structures are described here. Visualization of carbohydrate molecules in LiteMol has been updated by integration of the 3D implementation of the Symbol Nomenclature for Glycans (SNFG) system of carbohydrate representation (40,41) (Figure 4A). Fifty of the 71 monosaccharides defined by SNFG are present in the PDB in at least one anomeric form and are shown using 3D-SNFG symbols in LiteMol on the PDBe pages. This has simplified visualization and identification of carbohydrates, especially of complex and branched glycan chains.

From user testing it was clear that in the original implementation of LiteMol, the thin strands representing nucleic-acid backbones did not highlight these molecules sufficiently well. It is important that nucleic-acid chains be easily distinguishable from protein chains, and therefore a new visualization was developed for nucleic acids in LiteMol that addresses these problems and allows straightforward location of each base position (Figure 4B).

PDBe has worked with UniProt, Complex Portal (42), Reactome (43) and Ensembl (15) to support integration of LiteMol on their respective websites, placing the macromolecular structure within a context that is familiar to users of these resources. For example, Ensembl have implemented the display of variation data and exon boundaries directly on the macromolecular structure, helping researchers to more easily relate parts of the structure to genomic features.

## ACCESS TO EXPERIMENTAL DATA

The experimental data currently collected by wwPDB in OneDep (i.e., structure factors, chemical shifts and restraints, and electric potential maps) is a reduced (processed) representation of the raw experimental data. This enables computation of validation statistics, but to fully replicate the experiment, users require access to the raw experimental data which may have been deposited to specialized archives. PDBe previously introduced the Raw Experimental Data web component (19) to provide a summary and links to the raw experimental data from the Electron Microscopy Public Image Archive (EMPIAR) (44), SBGrid Data Bank (45) and Integrated Resource for Reproducibility in Macromolecular Crystallography (IRRMC) (46). Links to raw Free Induction Decay (FID) data held at BMRB for NMR entries have been added. The component relies on the respective archive to provide an API allowing queries based on PDB codes and serving basic information about, and a URL for, the raw experimental dataset. Independently, the OneDep system allows depositors to include a DOI to raw experimental data held in these or other archives. These DOIs have been standardized in OneDep and the component has been adapted to provide links to experimental datasets referenced by them.

## FURTHER VISUALIZATION IMPROVEMENTS

Improving the usability of PDBe services helps users in interpreting and exploiting the information provided, and a number of recent changes at PDBe have focused on improving the visualization experience. A specifically tailored mobile view has been developed for the PDBe search pages, which maintains the information provided on the desktop version but improves the usability on mobile devices. The use of web components across all PDBe pages gives an inherently more adaptable visualization, which has been utilized in the implementation of mobile-specific views.

The ProtVista component (47), developed in collaboration with UniProt and InterPro, enables a graphical visualization of molecular features corresponding to a protein's primary sequence. PDBe will in the near future include the ProtVista component on the PDB entry pages, where it will be interactively linked to a 2D topology component and the LiteMol viewer. The ProtVista implementation on these pages will display information related to the macromolecule in the PDB structure, including related functional data to provide further biological context.

## TRAINING AND OUTREACH

PDBe continues to run a broad programme of training and outreach activities to ensure that users can more easily utilize the tools and services to understand macromolecular structure data. These activities include webinars, workshops and seminars for researchers in the fields of chemistry, biology and bioinformatics. Since the beginning of 2018, PDBe have delivered 28 workshops in 13 different countries on 3 continents. The use of web-based components on the PDBe website enables easy delivery of workshops to a range of users without additional software requirements for the host or attendees. In addition, where users cannot attend PDBe workshops, training materials are accessible through the EMBL-EBI Train online portal (www.ebi.ac.uk/training/online) and the PDBe YouTube channel (youtube.com/proteindatabank). There have been 10 new videos added to the PDBe YouTube channel since the beginning of 2018, with videos on the PDBe YouTube channel viewed more than 15,000 times over the same time period.

The PDB Art project (pdbe.org/art) is a public engagement effort in collaboration with art societies and local schools. It engages students and the general public with macromolecular structure data through the medium of art. Students learn about protein structures in the PDB and the biology behind them, create artworks inspired by structures of their choice, and exhibit these works in a public art exhibition in Cambridge, UK. This project is now in its fifth year and has engaged hundreds of students and reached thousands of members of the general public. This project has been extremely valuable in communicating the importance of macromolecular structures with the general public and we encourage more scientists to use this approach, and to contact us for more information.

## FUTURE DEVELOPMENTS

PDBe will continue to improve the quality of data, aiming to improve findability through both the PDBe search facility and its REST API. This will include the integration of more data from the PDBe-KB resource into the search and entry pages, enriching the data further and improving navigation to relevant PDB entry-specific pages and the aggregated views offered by PDBe-KB. There will also be a continued effort to better classify and enrich data related to nucleic acids, as well as improved naming and classification of antibodies to help users more easily locate data for these molecules. In collaboration with Complex Portal (42), PDBe is working to classify known macromolecular complexes, which will enable standardization of assembly information available in the PDB, improving findability and further enriching data related to these assemblies.

The PDB archive continues to be a vital resource to drive the development of scientific research, with the tools provided by PDBe supporting a wide variety of users in making the most of these data. PDBe will continue to develop these tools and services for its continuously growing user community.

## Notes

Present address: Alice R. Clark, Faculty of Science and Engineering, University of Wolverhampton, Wulfruna Street, Wolverhampton WV1 1LY, UK.

Present address: Saqib Mir, GSK, Gunnels Wood Road, Stevenage, Herts SG1 2NY, UK.

Present address: Abhik Mukhopadhyay, The Cambridge Crystallographic Data Centre, 12 Union Road, Cambridge CB2 1EZ, UK.

Present address: Oliver Smart, School of Life Sciences, Anglia Ruskin University, East Road, Cambridge CB1 1PT, UK.

Present address: Hossam Zaki, Department of Molecular and Cellular Biology and Biochemistry, Brown University, Providence, RI, USA.
